# The effects of powered ankle-foot orthoses on joint kinematics and muscle activation during walking in individuals with incomplete spinal cord injury

**DOI:** 10.1186/1743-0003-3-3

**Published:** 2006-02-28

**Authors:** Gregory S Sawicki, Antoinette Domingo, Daniel P Ferris

**Affiliations:** 1 Division of Kinesiology, University of Michigan, Ann Arbor, MI, USA; 2 Department of Mechanical Engineering, University of Michigan, Ann Arbor, MI, USA; 3 Department of Biomedical Engineering, University of Michigan, Ann Arbor, MI, USA; 4 Department of Physical Medicine and Rehabilitation, Ann Arbor, USA

## Abstract

**Background:**

Powered lower limb orthoses could reduce therapist labor during gait rehabilitation after neurological injury. However, it is not clear how patients respond to powered assistance during stepping. Patients might allow the orthoses to drive the movement pattern and reduce their muscle activation. The goal of this study was to test the effects of robotic assistance in subjects with incomplete spinal cord injury using pneumatically powered ankle-foot orthoses.

**Methods:**

Five individuals with chronic incomplete spinal cord injury (ASIA C-D) participated in the study. Each subject was fitted with bilateral ankle-foot orthoses equipped with artificial pneumatic muscles to power ankle plantar flexion. Subjects walked on a treadmill with partial bodyweight support at four speeds (0.36, 0.54, 0.72 and 0.89 m/s) under three conditions: without wearing orthoses, wearing orthoses unpowered (passively), and wearing orthoses activated under pushbutton control by a physical therapist. Subjects also attempted a fourth condition wearing orthoses activated under pushbutton control by them. We measured joint angles, electromyography, and orthoses torque assistance.

**Results:**

A therapist quickly learned to activate the artificial pneumatic muscles using the pushbuttons with the appropriate amplitude and timing. The powered orthoses provided ~50% of peak ankle torque. Ankle angle at stance push-off increased when subjects walked with powered orthoses versus when they walked with passive-orthoses (ANOVA, p < 0.05). Ankle muscle activation amplitudes were similar for powered and passive-orthoses conditions except for the soleus (~13% lower for powered condition; p < 0.05).

Two of the five subjects were able to control the orthoses themselves using the pushbuttons. The other three subjects found it too difficult to coordinate pushbutton timing. Orthoses assistance and maximum ankle angle at push-off were smaller when the subject controlled the orthoses compared to when the therapist-controlled the orthoses (p < 0.05). Muscle activation amplitudes were similar between the two powered conditions except for tibialis anterior (~31% lower for therapist-controlled; p < 0.05).

**Conclusion:**

Mechanical assistance from powered ankle-foot orthoses improved ankle push-off kinematics without substantially reducing muscle activation during walking in subjects with incomplete spinal cord injury. These results suggest that robotic plantar flexion assistance could be used during gait rehabilitation without promoting patient passivity.

## Background

Motor recovery after neurological injury largely depends on maximizing neural plasticity [1,2]. The degree of functional neural plasticity is highly influenced by the amount of neural activity during rehabilitation. Passive, imposed movements can promote activity in sensory pathways but may not promote activity in motor pathways. Active movements require voluntary neuromuscular recruitment resulting in simultaneous activation of both efferent motor pathways and afferent sensory pathways. Training that emphasizes voluntary, active movements is much more effective at enhancing plasticity and increasing motor performance compared to training that emphasizes passive, imposed movements [3-5]. Repetitive active practice strengthens neural connections involved in a motor task through reinforcement learning. Practice is most effective when it is task-specific [6,7]. Thus, rehabilitation after neurological injury should emphasize repetitive, task-specific practice that promotes active neuromuscular recruitment in order to maximize motor recovery.

Locomotor training (or bodyweight supported treadmill training) is a gait rehabilitation method that aims to maximize activity-dependent plasticity. This technique was motivated by studies on the recovery of neural control of walking in spinalized cats. Spinal cats can re-learn to walk in response to repetitive step training on a treadmill [8-10]. Similar ideas have been extended to humans with neurological injury. The patient wears a harness that provides partial bodyweight unloading while they practice stepping on a treadmill. A team of physical therapists gives manual assistance to guide the lower limbs through a normal kinematic pattern [11]. To ensure task-specificity of the practice, therapists focus on providing rhythmic kinetic and kinematic sensory cues that are characteristic of healthy walking. Rhythmic limb loading [12], hip extension at the end of the stance phase [13], and the combination of contralateral limb movements with ipsilateral limb loading [14] all play some role in altering the motor output of spinal motor neuron pools. To encourage active patient effort, therapists provide manual assistance only 'as needed'. One long-term study reported that 80% of wheelchair bound patients with chronic incomplete spinal cord injury gained functional walking ability after treadmill training with partial bodyweight support and therapist manual assistance [15]. Locomotor training is a promising therapy for patients with neurological injury but places a considerable burden on the therapists who must administer the manual assistance.

Recent progress in rehabilitation robotics has resulted in machines that can effectively automate therapist manual assistance during locomotor training [16]. The Mechanized Gait Trainer [17,18], Lokomat^® ^[19,20] and PAM, POGO and ARTHuR [21] are all examples of robotic devices that are integrated into a treadmill and bodyweight support system in order to assist stepping. Each of these devices can actively assist the patient's limbs, guiding them through a pre-programmed physiological gait pattern by driving the hip and knee. These robotic devices make it possible for a single therapist to administer locomotor training with little physical labor because the device provides the mechanical assistance. These large, stationary devices make the job of the therapist easier but they may encourage passivity by the patient during locomotor training. Another drawback to these devices is that they only assist the hip and knee.

The ankle joint plays an important role in the mechanics and neural control of walking. The ankle plantar flexors provide ~70% of the joint work during walking, far more than the muscles crossing the hip or knee [22,23]. The muscles acting at the ankle joint act to support the body, propel the center of mass forward during push-off [24,25] and reduce energy losses due to the plastic collision of the leading leg at heel strike [26]. In addition, feedback from ankle joint afferents is critical to the neural control of walking [27-30]. Individuals with incomplete spinal cord injury typically exhibit abnormal ankle kinematics and deficits in top speed during walking due to lack of propulsion [31]. Because of its relative importance to the mechanics, energetics and control of walking gait, providing active assistance at the ankle joint during locomotor training may be important.

Few studies have examined the effect of mechanical assistance during locomotor training on lower limb kinematics and muscle activation patterns of patients with spinal cord injury. Two groups reported that healthy subjects alter muscle activation patterns for walking in the Lokomat^® ^compared to unassisted walking [32,33] but did not test neurologically impaired subjects. Hornby et al. [34] and Colombo et al. [35] examined individuals with spinal cord injury and found differences in muscle activation patterns between stepping with Lokomat^® ^and stepping with manual assistance. Both studies found that individuals with incomplete spinal cord injury have lower muscle activation amplitudes with Lokomat^® ^assistance compared to manual assistance. Hornby et al. [34] also provided data that subjects have 40% lower oxygen consumption during stepping with Lokomat^® ^assistance compared to stepping with manual assistance. A more thorough understanding of how mechanical assistance alters muscle activation patterns and kinematics in neurologically impaired subjects is important for development of more effective rehabilitation robotic devices and strategies.

The goal of this study was to examine the effect of robotic plantar flexion assistance on the muscle activation and kinematic patterns of walking in subjects with incomplete spinal cord injury. To study these effects we built wearable, powered ankle-foot orthoses [36,37]. The orthoses were lightweight, strong and custom fitted to each subject. Pneumatic actuators powered ankle plantar flexion [38-40]. Hand-held pushbuttons allowed a therapist or the subject to control the timing and magnitude of orthoses assistance. We hypothesized that powered plantar flexor assistance would (1) lead to increased plantar flexion at push-off and (2) reduce neuromuscular recruitment of the triceps surae group (soleus, medial gastrocnemius and lateral gastrocnemius).

## Methods

We recruited two males and three females (height 170.7 ± 10.9 cm; body mass 86.3 ± 22.6 kg; 44.6 ± 13.4 years of age; mean ± SD) with chronic incomplete spinal cord injury at the cervical or thoracic level (ASIA C-D). Participants were required to be greater than 18 years of age, more than 6 months post injury with no history of orthopedic complications, and to have limited walking ability (see Table 1 for details). A physician examined and cleared each subject for participation. Subjects read and signed a consent form prepared according to the Declaration of Helsinki and approved by the University of Michigan Medical School Institution Review Board for Human Subject Research.

**Table 1:** Subject Information. Data for each subject that describe age, body size, injury level, and walking ability.

**Subject**	**Age (yrs.)**	**Sex** **Height (cm)** **Weight (kg)**	**Injury Etiology**	**Injury Level**	ASIA* Level	**Post Injury (mos.)**	**Walking Aids**	**Overground Speed (m/s)**	**BWS Level (%)** **Speeds (m/s)** **Active** **Orthoses Conditions**
**1**	54	F 165.1 cm 73.7 kg	Dermoid Tumor	T11/T12	C	64	Cane (L,R) Orthosis (L)	0.41	50% 0.36–0.89 TC,PC
**2**	52	F 156.2 cm 58.1 kg	Myxopapillary Ependymoma	T8/L2	D	93	Cane (R)	0.61	30% 0.36–0.89 TC
**3**	38	F 175.3 cm 115.3 kg	Transverse Myelitis	T5	D	77	Cane (R) Orthosis (L)	0.37	50% 0.36–0.89 TC
**4**	24	M 185.4 cm 101.5 kg	Trauma	T10/T11	D	111	_	0.95	30% 0.36–0.89 TC,PC
**5**	55	M 171.5 cm 83.0 kg	Sarcoidosis	C5/C6	C	144	Cane (R)	0.48	30% 0.36–0.54 TC

* ASIA = American Spinal Injury Association Impairment Scale A = complete E=normal

**Figure 1:**
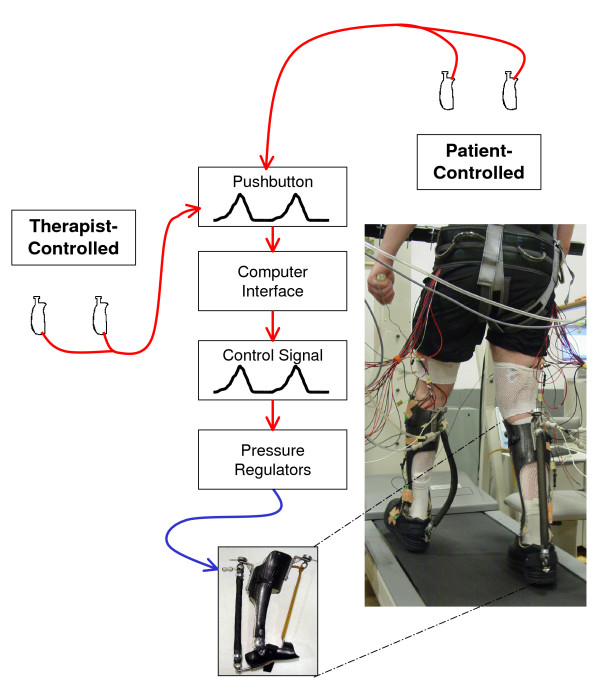
**University of Michigan Powered Ankle-Foot Orthosis**. Schematic shows signal flow from hand-held pushbuttons activated *either *by a therapist *or *by the patient. The pushbuttons generate a real-time voltage proportional to the amount of button press. A computer interface converts this voltage to a control signal (0–10 V). The control signal activates solenoid gated pressure valves that regulate the flow of air into and out of artificial pneumatic muscles on the lightweight carbon fiber ankle-foot orthoses. A 24 year old male (ASIA D) practices walking on a treadmill with partial bodyweight support using the hand-held pushbuttons to command plantar flexor torque assistance at his ankles (right).

We custom fitted each subject with bilateral ankle-foot orthoses (Figure 1). Details of the orthosis design have been described previously [38-40]. Each orthosis consisted of an ankle hinge joint connecting a carbon fiber shank section and a polypropylene foot section. The orthoses constrained ankle rotation to the sagittal plane. We attached a single artificial pneumatic actuator between two metal brackets on the posterior of each orthosis to provide powered ankle plantar flexion during walking. We also attached an elastic cord between brackets on the anterior of each orthosis to prevent toe drag. A load transducer (LC8150-375-1K 0–100 lbs, Omega Engineering, Inc., Stamford, CT) in series with each artificial muscle monitored the tension that the actuator produced during walking. Each orthosis weighed 1.09 ± 0.15 kg and had an average extensor moment arm of 9.7 ± 1.2 cm, flexor moment arm of 10.0 ± 1.1 cm and artificial muscle length of 43.3 ± 4.0 cm (all mean ± SD). Four parallel proportional pressure regulators (valve PPC0445A-ACA-OAGABA09 and solenoid 45A-L00_DGFK-1BA, MAC Valves, Inc. Wixom, MI) supplied compressed air to each artificial muscle via nylon tubing (0–6.2 bar). Analog-controlled solenoid valves in parallel with the air supply tubing improved exhaust dynamics (35A-AAA-0DAJ-2KJ, MAC Valves, Inc., Wixom, MI).

We used a real-time computer interface (dSPACE Inc., Northville, MI; 1000 Hz sampling) to control the air pressure supplied to the artificial pneumatic muscles based on a signal generated from a pushbutton held in each hand. When the pushbutton plunger was fully depressed, a control signal (10 V) was sent to the pressure regulators to command maximal air pressure to the artificial pneumatic muscle. When the pushbutton plunger was not depressed, no control signal (0 V) was generated and no air pressure was supplied to the muscle. We programmed the controller to exhibit linear behavior proportional to the displacement of the plunger between no air pressure and maximum air pressure. The time between the control signal onset and initial rise of artificial muscle tension (~50 ms) of the device is comparable to response times of human plantar flexors and should not cause compensatory strategies by the user [38]. The pushbutton controllers could be operated by a therapist administering training or by the subject (Figure 1).

Subjects completed two testing sessions. The first day was a practice session used to assess the required bodyweight support level and speed capability for each subject. It also provided a chance for the participants to become acclimated to wearing the powered orthoses during locomotor training. A typical practice session allowed 10–15 minutes of stepping with the orthoses in each condition (total 30–45 minutes of stepping). Breaks were given after each bout of stepping or when the subject requested a rest. Prior to therapist-controlled and patient-controlled conditions we informed the therapist and patients that the assistance was proportional to the pushbutton plunger displacement but gave no explicit instructions about how much they should depress the plungers. If needed, some instruction was given to the subject to help with the timing of the pushbutton activation during the patient-controlled conditions. This was done by using verbal cues (eg. "now", "now") to help them find an appropriate pattern. The time between the first and second session varied between subjects from 10–34 days.

On the second day data was acquired while subjects completed walking trials on a treadmill with a set level of partial bodyweight support at four speeds (0.36, 0.54, 0.72 and 0.89 m/s) under three conditions per speed: (1) without wearing orthoses (without-orthoses, **WO**) (2) wearing bilateral orthoses unpowered (passive-orthoses, **PA**) and (3) wearing bilateral orthoses powered under pushbutton control by a therapist (therapist-controlled, **TC**). Two subjects completed a fourth condition (4) wearing bilateral orthoses powered under pushbutton control by the subject her/himself (patient-controlled, **PC**). One subject could not complete the 0.72 m/s and 0.89 m/s speeds for all conditions. Subjects were not blinded to experimental conditions and given time to re-acclimate themselves with each experimental condition before data was acquired. Verbal cues to assist timing were not given during data collection periods. Subjects wore their own athletic shoes for the without-orthoses condition and commercially available orthoses shoes for all other conditions. Heel heights were similar and should not have affected the results. Partial unloading was provided with a bodyweight support system (Robomedica Inc., Pasadena, CA). The subjects wore a modified parachute harness around the trunk that was attached to a cable supplying a load to offset part of bodyweight. A feedback controller and pneumatic actuator enforced the desired level of unloading. Unloading level was set to either 30% (subject supports 70% of his/her weight) or 50% (subject supports 50% of her/his weight) depending on walking ability. The bodyweight support level was constant across the session for each individual. Elastic cords provided lateral stabilization. Trials were pseudo-randomized to eliminate ordering effects. Breaks were given after each bout of stepping or when the subject requested a rest. Breaks varied in length but were typically never longer than 3–5 minutes.

At the beginning of the practice session (day 1) subjects walked overground with their normal aids (canes, braces, walkers) so we could record the preferred walking speed. On day two, during treadmill walking trials, we recorded two 10-second intervals of bilateral joint angles and foot-ground contact, lower limb surface electromyography, pushbutton control signal, artificial muscle force and elastic band force. We recorded bilateral ankle, knee and hip joint angles using electrogoniometers (1200 Hz, Biometrics, Ltd., Ladysmith, VA). Goniometers were re-zeroed in the neutral position before each condition. We recorded stride cycle data from each foot using a pair of complete footswitches (B & L Engineering, Tustin CA). We recorded bilateral lower limb surface electromyography (EMG) (1200 Hz, Konigsberg Instruments, Inc., Pasadena, CA) of tibialis anterior (TA), soleus (SOL), medial gastrocnemius (MG), lateral gastrocnemius (LG), vastus medialis (VM), vastus lateralis (VL), rectus femoris (RF) and medial hamstrings (MH) using bipolar surface electrodes (2.5 cm inter-electrode distance). The EMG amplifier bandwidth was 1000 Hz. We visually inspected EMG during manual muscle tests prior to walking to minimize cross talk, moving electrode placements as necessary. We recorded artificial pneumatic muscle and elastic band tension using tension/compression force transducers (1200 Hz, Omega Engineering, Stamford, CT) placed in series with the orthoses attachment brackets. All signals were collected simultaneously via the same data acquisition board to ensure synchronization.

**Figure 2:**
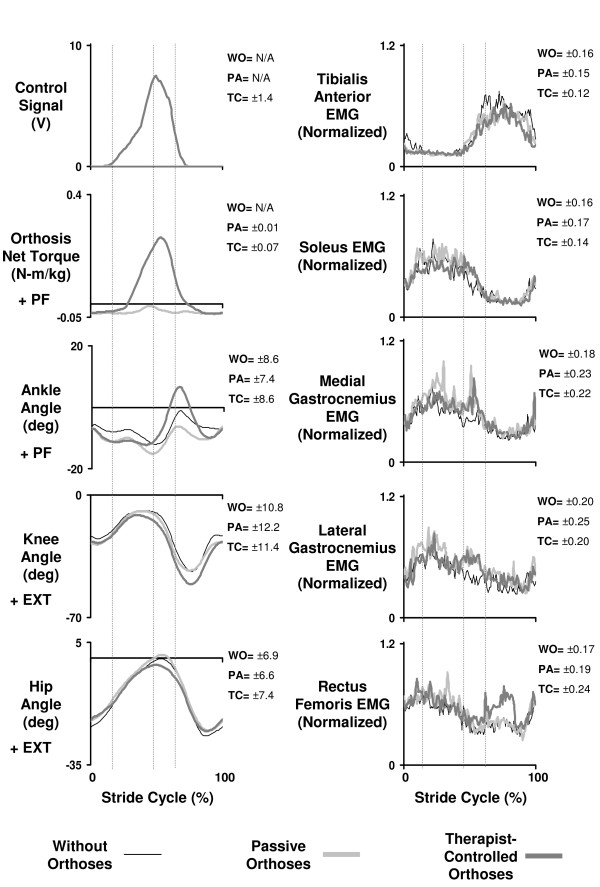
**Kinematics, kinetics and electromyography for without vs. passive vs. therapist-controlled-orthoses**. Mean data for five subjects with incomplete spinal cord injury who walked with partial bodyweight support on a treadmill at 0.54 m/s while wearing no orthoses (without-orthoses), wearing orthoses unpowered (passive-orthoses) and wearing orthoses powered under pushbutton control by a therapist (therapist-controlled orthoses). Stride cycles begin (0%) and end (100%) at heel strike. Double support phases are indicated by vertical lines. The average standard deviation over the stride cycle for each signal and each condition is reported to the right of each plot in units consistent with that signal.

We formed average stride cycle profiles for EMG, kinematic and kinetic variables for each subject using the software package Visual 3D (C-Motion Inc., Rockville, MD). Average stride cycle profiles were calculated from heel strike to heel strike of the right and left leg using foot contact information from foot switches. All complete stride cycles occurring for the right and left leg during each of two 10-second trials for each experimental condition were used to form the average stride cycle profile. The number of complete stride cycles captured ranged from 8 to 14 strides depending on the trial speed, trial condition and fidelity of the data. We calculated the average standard deviation over the stride cycle (reported in Figures 2 and 5) for each average profile and for each condition to quantify the variability in the data.

EMG data were filtered using a zero-lag fourth-order Butterworth high pass filter (cutoff frequency 20 Hz) and then full wave rectified. The stride cycle averaged EMG data was normalized to the maximum value of the average stride cycle profile during the without-orthoses condition at 0.54 m/s for each muscle. To examine changes in EMG amplitude across conditions, normalized average root mean square (RMS) EMG values were calculated for each subject for each condition and speed combination. Average RMS EMG values were calculated for the total, stance and swing phases of the gait cycle separately. RMS window sizes were chosen to match the length of the cycle of interest and a single average value was computed for each interval. Average RMS EMG values were normalized to the maximum value of the average RMS EMG value for the without-orthoses condition at 0.54 m/s for each muscle.

We also created stride cycle profiles for joint angle data created from smoothed goniometer data (low pass filtered, cutoff frequency 6 Hz). To examine changes in kinematics across conditions, we calculated the joint range of motion for the ankle, knee and hip over the gait cycle. In addition, because our assistance focused on creating improved ankle push-off kinematics, we measured the maximum ankle angle over the gait cycle. We also calculated the total gait cycle duration, stance phase duration, swing phase duration and double support phase duration. We created stride cycle control signal profiles from the recorded pushbutton signal input and stride cycle orthoses torque profiles from the artificial muscle and elastic band tension and their respective moment arms. The orthoses torque was normalized to subject mass. To quantify the magnitude and repeatability of the control signal generated by the user (therapist/patient) we calculated the maximum control signal achieved over the stride cycle. To quantify the level of mechanical assistance of the powered orthoses, we calculated the maximum orthoses torque over the gait cycle. Finally, to examine differences in the timing of assistance between the therapist-controlled and patient-controlled conditions, we calculated the onset of the control signal and the onset of orthoses plantar flexor torque (i.e. > 0) as a percentage of the gait cycle.

We used separate repeated measures three-way (by subject, condition and speed) analysis of variance tests (ANOVAs) to test for differences in maximum ankle extension angle, ankle, knee and hip range of motion and normalized stance phase RMS EMG for the muscles of the lower leg between conditions (WO, PA, TC) for all five subjects (JMP IN software, SAS Institute, Inc.). We also calculated an interaction effect between speed and condition for ankle range of motion and maximum ankle angle. We carried out the same procedure to test for differences between active conditions (TC; PC) for the two subjects that could complete the PC condition. We set the significance level at p < 0.05 and used Tukey Honestly Significant Difference (THSD) post-hoc tests where appropriate. Finally we calculated statistical power for each test.

Some data were not included in the average step cycle profiles, metric calculations and statistical analysis. Recall that only four of the five subjects could complete trials at 0.72 m/s and 0.89 m/s. Due to the tight fit of the orthoses over the lower limbs we lost the TA EMG for one subject. Two subjects had very low EMG activity in one leg due to the severity of their injury. For those two subjects we used only the more active leg to compute subject averages. In addition, for one subject we could not calculate double support duration because of a damaged footswitch.

## Results

Subjects' preferred overground walking speed with their walking aids was 0.56 ± 0.10 m/s (all data reported are mean ± SE). Table 1 indicates the speeds and walking aids used for each subject. Four of the five subjects exceeded their preferred overground walking speed when walking at their top treadmill speed.

The data that follows in the results section are from the second day of testing after the training session was completed (see Methods). With the exception of ankle joint kinematics, all differences in conditions showed similar trends across speeds. Therefore, data reported in the text are averaged by condition across subjects and speeds unless otherwise noted. In addition, data averaged by speed and by condition across subjects are reported in Tables 2, 3, 4, 5.

**Table 2:** Kinematics for without, passive, and therapist-controlled orthoses by speed. Mean ± standard error and statistical results for kinematics of subjects with incomplete spinal cord injury who walked without-orthoses (WO), wearing orthoses unpowered or passive (PA) and wearing orthoses powered under pushbutton control by a therapist (TC) for 0.36 m/s (five subjects), 0.54 m/s (five subjects), 0.72 m/s (four subjects) and 0.89 m/s (four subjects).

	**ANOVA p-value**	**THSD**	**0.36 m/s**			**0.54 m/s**			**0.72 m/s**			**0.89 m/s**		
	
			***WO***	***PA***	***TC***	***WO***	***PA***	***TC***	***WO***	***PA***	***TC***	***WO***	***PA***	***TC***
			
**Ankle ROM (deg)**			17.3 ± 4.0	16.5 ± 2.5	31.2 ± 3.5	19.8 ± 5.1	18.9 ± 2.5	29.9 ± 1.8	25.6 ± 6.9	21.7 ± 3.6	27.7 ± 3.5	25.7 ± 7.3	23.7 ± 3.9	25.9 ± 2.7
**Max Ankle (deg)**	<0.0001 * P = 1.00	TC > PA TC > WO PA < WO	2.8 ± 4.2	-1.0 ± 3.9	13.5 ± 3.9	4.0 ± 4.2	0.72 ± 3.9	12.0 ± 3.7	10.7 ± 4.4	4.4 ± 3.4	11.8 ± 2.6	12.0 ± 5.6	6.8 ± 3.5	10.3 ± 1.7
**Knee ROM (deg)**	0.4136 P = 0.20		44.4 ± 8.8	43.3 ± 8.7	47.3 ± 8.7	44.3 ± 9.7	47.9 ± 7.1	49.4 ± 6.6	52.6 ± 9.1	53.0 ± 5.9	53.6 ± 5.0	53.5 ± 8.7	54.1 ± 5.1	52.9 ± 4.1
**Hip ROM (deg)**	<0.0001 * P = 1.00	TC < PA TC < WO	25.6 ± 3.4	24.6 ± 3.5	23.2 ± 2.8	28.4 ± 3.1	28.2 ± 2.7	23.9 ± 2.9	31.5 ± 4.0	28.7 ± 3.5	24.3 ± 3.3	33.6 ± 3.3	32.0 ± 3.6	26.4 ± 3.5
**Total Time (s)**	0.2360 P = 0.30		1.98 ± 0.24	1.92 ± 0.22	1.88 ± 0.14	1.65 ± 0.17	1.62 ± 0.15	1.54 ± 0.13	1.57 ± 0.15	1.49 ± 0.13	1.42 ± 0.09	1.31 ± 0.08	1.35 ± 0.11	1.34 ± 0.07
**Stance Time (s)**	0.7611 P = 0.10		1.26 ± 0.19	1.18 ± 0.15	1.26 ± 0.15	1.01 ± 0.11	0.98 ± 0.10	0.99 ± 0.10	0.92 ± 0.09	0.88 ± 0.07	0.87 ± 0.08	0.76 ± 0.03	0.80 ± 0.07	0.74 ± 0.05
**Swing Time (s)**	0.0643 P = 0.54		0.72 ± 0.06	0.74 ± 0.09	0.61 ± 0.07	0.65 ± 0.06	0.63 ± 0.06	0.56 ± 0.05	0.65 ± 0.06	0.62 ± 0.06	0.56 ± 0.04	0.53 ± 0.06	0.55 ± 0.06	0.60 ± 0.07
**Double Support Time (s)**	0.0173 * P = 0.74	TC > WO	0.27 ± 0.12	0.27 ± 0.06	0.43 ± 0.15	0.18 ± 0.06	0.22 ± 0.05	0.25 ± 0.06	0.13 ± 0.03	0.15 ± 0.04	0.17 ± 0.04	0.06 ± 0.04	0.14 ± 0.04	0.14 ± 0.01

Values are means ± SE. See METHODS for calculations.

* Indicates a p-value of less than 0.05 showing significant differences between conditions. Statistical power, P, is reported under the p-value. Tukey Honestly Significant Difference, THSD, results are reported for metrics with significance.

Five subjects completed all conditions at 0.36 m/s and 0.54 m/s. Four subjects completed all conditions at 0.72 m/s and 0.89 m/s.

Double support time is for four subjects for all conditions at all speeds.

**Table 3:** Stance RMS EMG for without, passive and therapist-controlled orthoses by speed. Mean ± standard error and statistical results for the normalized average root mean square muscle activation calculated from the stance phase electromyography records for: tibialis anterior (TA), soleus (SOL), medial gastrocnemius (MG), lateral gastrocnemius (LG), vastus medialis (VM), vastus lateralis (VL), rectus femoris (RF) and medial hamstrings (MH). Subjects with partial paralysis walked without-orthoses (WO), wearing orthoses unpowered or passive (PA) and wearing orthoses powered under pushbutton control by a therapist (TC) at 0.36 m/s (five subjects), 0.54 m/s (five subjects), 0.72 m/s (four subjects) and 0.89 m/s (four subjects). TA is for four subjects at all speeds. Stance phase root mean square EMG was normalized to the without condition at 0.54 m/s for each muscle.

	**ANOVA p-value**	**THSD**	**0.36 m/s**			**0.54 m/s**			**0.72 m/s**			**0.89 m/s**		
	
			***WO***	***PA***	***TC***	***WO***	***PA***	***TC***	***WO***	***PA***	***TC***	***WO***	***PA***	***TC***
			
**TA**	0.0845 P = 0.49		0.87 ± 0.16	0.72 ± 0.16	0.75 ± 0.10	0.89 ± 0.02	0.75 ± 0.12	0.81 ± 0.14	1.10 ± 0.12	0.99 ± 0.12	0.80 ± 0.16	1.04 ± 0.10	1.07 ± 0.04	0.79 ± 0.15
**SOL**	0.0197 * P = 0.72	TC < PA	0.81 ± 0.08	0.94 ± 0.06	0.80 ± 0.05	0.95 ± 0.02	1.07 ± 0.06	0.87 ± 0.07	1.06 ± 0.08	1.07 ± 0.06	0.99 ± 0.07	1.14 ± 0.10	1.27 ± 0.15	1.12 ± 0.06
**MG**	0.0229 * P = 0.70	PA > WO	0.70 ± 0.11	0.87 ± 0.11	0.79 ± 0.13	0.92 ± 0.02	1.12 ± 0.08	1.00 ± 0.07	1.03 ± 0.10	1.14 ± 0.10	1.08 ± 0.10	1.12 ± 0.13	1.34 ± 0.19	1.27 ± 0.13
**LG**	0.0436 * P = 0.61	PA > WO	0.79 ± 0.09	0.91 ± 0.08	0.83 ± 0.10	0.93 ± 0.01	1.08 ± 0.08	0.98 ± 0.07	1.03 ± 0.10	1.15 ± 0.02	1.12 ± 0.06	1.18 ± 0.07	1.35 ± 0.09	1.35 ± 0.14
**VM**	0.0145 * P = 0.76	PA > WO	0.81 ± 0.05	0.94 ± 0.05	0.83 ± 0.04	0.97 ± 0.00	1.09 ± 0.07	1.08 ± 0.08	1.12 ± 0.05	1.16 ± 0.06	1.06 ± 0.07	1.16 ± 0.02	1.26 ± 0.05	1.14 ± 0.08
**VL**	0.0424 * P = 0.61	PA > WO	0.86 ± 0.03	0.95 ± 0.03	0.90 ± 0.06	0.96 ± 0.01	1.16 ± 0.09	1.05 ± 0.11	1.10 ± 0.04	1.18 ± 0.10	1.07 ± 0.14	1.12 ± 0.02	1.21 ± 0.08	1.16 ± 0.19
**RF**	0.0123 * P = 0.77	TC < PA PA > WO	0.85 ± 0.04	0.94 ± 0.03	0.93 ± 0.08	0.95 ± 0.01	1.13 ± 0.06	1.06 ± 0.09	1.17 ± 0.09	1.15 ± 0.06	1.01 ± 0.10	1.17 ± 0.03	1.30 ± 0.08	1.09 ± 0.13
**MH**	0.1954 P = 0.34		0.92 ± 0.03	0.86 ± 0.06	0.89 ± 0.07	0.92 ± 0.02	0.98 ± 0.04	1.02 ± 0.08	1.02 ± 0.07	1.03 ± 0.10	1.22 ± 0.18	1.03 ± 0.08	1.13 ± 0.13	1.19 ± 0.22

Values are means ± SE. Data are unitless because of normalization. See METHODS for calculations.

* Indicates a p-value of less than 0.05 showing significant differences between conditions. Statistical power, P, is reported under the p-value. Tukey Honestly Significant Difference, THSD, results are reported for metrics with significance.

Five subjects completed all conditions at 0.36 m/s and 0.54 m/s. Four subjects completed all conditions at 0.72 m/s and 0.89 m/s.

TA is for four subjects for all conditions at all speeds.

**Table 4:** Kinematics for therapist-controlled and patient-controlled orthoses by speed. Mean ± standard error and statistical results for kinematics of two subjects with incomplete spinal cord injury who walked wearing orthoses powered under pushbutton control by a therapist (TC) and wearing orthoses powered under pushbutton control by the patient him/herself (PC) for 0.36 m/s, 0.54 m/s, 0.72 m/s and 0.89 m/s.

	**ANOVA p-value**	**THSD**	**0.36 m/s**		**0.54 m/s**		**0.72 m/s**		**0.89 m/s**	
	
			***TC***	***PC***	***TC***	***PC***	***TC***	***PC***	***TC***	***PC***
			
**Ankle ROM (deg)**	0.1613 P = 0.28		25.8 ± 2.9	22.3 ± 2.2	29.4 ± 1.3	24.0 ± 7.5	23.1 ± 3.1	20.5 ± 3.0	22.1 ± 3.6	23.7 ± 2.4
**Max Ankle (deg)**	0.0224 * P = 0.68	PC < TC	13.2 ± 0.9	8.5 ± 0.5	15.5 ± 5.9	7.8 ± 2.9	9.5 ± 2.6	3.9 ± 1.3	8.7 ± 2.1	7.5 ± 0.3
**Knee ROM (deg)**	0.3591 P = 0.14		52.9 ± 5.7	55.4 ± 7.4	55.5 ± 5.1	54.1 ± 8.7	56.6 ± 4.7	57.3 ± 5.1	55.8 ± 3.9	58.3 ± 7.4
**Hip ROM (deg)**	0.1172 P = 0.34		18.2 ± 5.8	23.3 ± 13.7	18.6 ± 5.4	24.3 ± 12.8	21.2 ± 6.7	24.8 ± 14.5	22.4 ± 5.6	26.3 ± 15.4
**Total Time (s)**	0.0631 P = 0.47		1.71 ± 0.14	1.67 ± 0.24	1.46 ± 0.13	1.42 ± 0.12	1.31 ± 0.05	1.24 ± 0.10	1.31 ± 0.15	1.15 ± 0.05
**Stance Time (s)**	0.0145 * P = 0.76	PC < TC	1.20 ± 0.01	1.08 ± 0.08	0.93 ± 0.01	0.90 ± 0.00	0.80 ± 0.01	0.73 ± 0.00	0.70 ± 0.03	0.67 ± 0.01
**Swing Time (s)**	0.5458 P = 0.09		0.50 ± 0.12	0.57 ± 0.15	0.53 ± 0.12	0.52 ± 0.11	0.51 ± 0.06	0.50 ± 0.10	0.62 ± 0.17	0.48 ± 0.06
**Double Support Time (s)**	N/A		0.41 ± 0.00	0.29 ± 0.00	0.26 ± 0.00	0.24 ± 0.00	0.18 ± 0.00	0.17 ± 0.00	0.14 ± 0.00	0.13 ± 0.00

Values are means ± SE. See METHODS for calculations.

* Indicates a p-value of less than 0.05 showing significant differences between conditions. Statistical power, P, is reported under the p-value. Tukey Honestly Significant Difference, THSD, results are reported for metrics with significance.

Two subjects completed all conditions at all speeds.

Double support time is for a single subject. As a result no statistical tests could be carried out for this metric.

**Table 5:** Stance RMS EMG for therapist-controlled and patient-controlled orthoses by speed. Mean ± standard error and statistical results for the normalized average root mean square muscle activation calculated from the stance phase electromyography records for: tibialis anterior (TA), soleus (SOL), medial gastrocnemius (MG), lateral gastrocnemius (LG), vastus medialis (VM), vastus lateralis (VL), rectus femoris (RF) and medial hamstrings (MH). Two subjects with partial paralysis walked with orthoses powered under pushbutton control by a therapist (TC) and with orthoses powered under pushbutton control by the patient him/herself (PC) at 0.36 m/s, 0.54 m/s, 0.72 m/s and 0.89 m/s. Stance phase root mean square EMG was normalized to the without condition at 0.54 m/s for each muscle.

	**ANOVA p-value**	**THSD**	**0.36 m/s**		**0.54 m/s**		**0.72 m/s**		**0.89 m/s**	
	
			***TC***	***PC***	***TC***	***PC***	***TC***	***PC***	***TC***	***PC***
			
**TA**	0.0090 * P = 0.83	PC > TC	0.61 ± 0.16	0.89 ± 0.54	0.71 ± 0.32	1.01 ± 0.58	0.76 ± 0.27	1.08 ± 0.41	0.66 ± 0.14	1.04 ± 0.45
**SOL**	0.4801 P = 0.10		0.85 ± 0.16	0.73 ± 0.33	0.80 ± 0.13	0.78 ± 0.40	0.99 ± 0.16	0.94 ± 0.52	1.13 ± 0.09	1.03 ± 0.49
**MG**	0.7697 P = 0.06		0.95 ± 0.09	0.92 ± 0.20	0.97 ± 0.01	1.03 ± 0.24	1.17 ± 0.04	1.16 ± 0.35	1.39 ± 0.10	1.26 ± 0.34
**LG**	0.7072 P = 0.06		0.93 ± 0.21	0.88 ± 0.22	0.95 ± 0.01	1.01 ± 0.28	1.20 ± 0.09	1.21 ± 0.44	1.53 ± 0.21	1.29 ± 0.42
**VM**	0.2861 P = 0.18		0.86 ± 0.01	0.97 ± 0.07	1.09 ± 0.10	0.77 ± 0.25	1.13 ± 0.07	1.17 ± 0.09	1.22 ± 0.13	1.02 ± 0.00
**VL**	0.1380 P = 0.31		0.95 ± 0.03	0.89 ± 0.04	0.95 ± 0.03	0.65 ± 0.16	0.98 ± 0.04	1.00 ± 0.16	0.98 ± 0.04	0.91 ± 0.06
**RF**	0.6351 P = 0.07		0.88 ± 0.06	0.82 ± 0.01	0.94 ± 0.03	0.63 ± 0.18	0.96 ± 0.00	1.12 ± 0.23	0.94 ± 0.05	0.97 ± 0.06
**MH**	0.3099 P = 0.16		1.01 ± 0.01	1.36 ± 0.35	1.17 ± 0.14	1.18 ± 0.68	1.43 ± 0.34	1.73 ± 0.48	1.48 ± 0.38	1.38 ± 0.31

Values are means ± SE. Data are unitless because of normalization. See METHODS for calculations.

* Indicates a p-value of less than 0.05 showing significant differences between conditions. Statistical power, P, is reported under the p-value. Tukey Honestly Significant Difference, THSD, results are reported for metrics with significance.

Two subjects completed all conditions at all speeds.

### Therapist-controlled vs. passive and without-orthoses

Within the thirty-minute practice session, the therapist was able to activate the hand-held pushbuttons to produce appropriate timing of powered assistance for all subjects. The therapist required only a few minutes of practice with some subjects while other subjects required a longer training period. In all cases, both the therapist and subject agreed that they established a consistent walking pattern by the end of the thirty-minute practice session.

The control signal generated by the therapist had an onset in early stance at 25.5 ± 3.3% of the gait cycle. Peak control signal activation was 8.8 ± 0.3 V (out of 10 V) and resulted in orthoses ankle plantar flexor torque onset at 34.2 ± 4.0% of the gait cycle. The powered orthoses applied 0.38 ± 0.03 N-m/kg peak ankle plantar flexion torque at the end of the stance phase (Figure 2).

Powered assistance under therapist control modified joint kinematics compared to the other conditions. Ankle joint range of motion was greater for the therapist-controlled orthoses condition compared to the passive-orthoses and without-orthoses conditions (ANOVA, p < 0.0001) (Figure 2, Table 2). Subjects achieved an ankle range of motion of 28.9 ± 1.4 degrees while walking with the orthoses providing torque assistance under therapist control. This was 9 degrees more than while walking with the orthoses passive and 7 degrees more than while walking without the orthoses. The improvement in ankle range of motion was mainly due to increased plantar flexion at push-off. In the therapist-controlled active condition the subjects walked with a maximum ankle angle at push-off of 12.0 ± 1.5 degrees. This was 9.6 degrees more than for walking with the orthoses passive and 5.1 degrees more than for walking without the orthoses.

**Figure 3:**
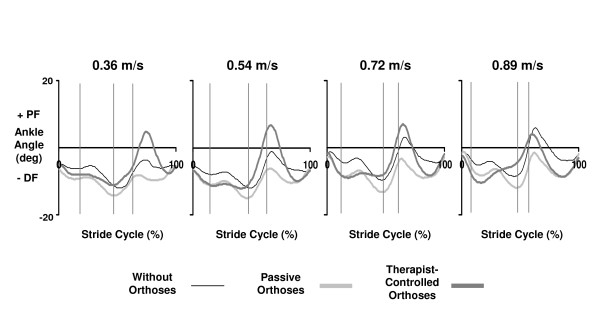
**Ankle kinematics for without vs. passive vs. therapist-controlled orthoses across speeds**. Mean ankle joint angle data for subjects with incomplete spinal cord injury who walked on a treadmill with partial bodyweight support at (left to right) 0.36 m/s (five subjects), 0.54 m/s (five subjects), 0.72 m/s (four subjects) and 0.89 m/s (four subjects). For each speed, subjects walked wearing no orthoses (without-orthoses), wearing orthoses unpowered (passive-orthoses) and wearing orthoses powered under pushbutton control by a therapist (therapist-controlled orthoses). Stride cycles begin (0%) and end (100%) at heel strike. Double support phases are indicated by vertical lines.

Improvements in ankle kinematics due to powered plantar flexion assistance were larger for slow walking speeds than for fast walking speeds (Figure 3, Table 2). There was a significant interaction between speed and condition for the maximum ankle angle at push-off (p = 0.02). At 0.54 m/s the maximum ankle angle was 11 degrees more in the therapist-controlled orthoses versus the passive-orthoses condition but at 0.89 m/s that difference was only 3.5 degrees.

Knee and hip joint kinematics were not modified as greatly as ankle joint kinematics in the powered orthoses condition. The knee joint range of motion was not significantly different between conditions or across speeds (p > 0.05). Powered torque assistance decreased hip joint range of motion slightly compared to the passive condition (p < 0.0001) (Figure 2, Table 2). When subjects walked with the orthoses passive the hip joint range of motion was 28.2 ± 1.6. When the subjects walked wearing the orthoses powered under therapist control the hip range of motion decreased by ~4 degrees to 24.4 ± 1.4 degrees.

Therapist-controlled powered ankle assistance significantly increased the time of double support when compared to the without-orthoses condition (p < 0.05) (Table 2). The average time for double support in the therapist-controlled orthoses condition was 88 ms longer than the without-orthoses condition and 55 ms longer than the passive-orthoses condition. The total, stance phase and swing phase average gait cycle durations were not significantly different between conditions (p > 0.05).

**Figure 4:**
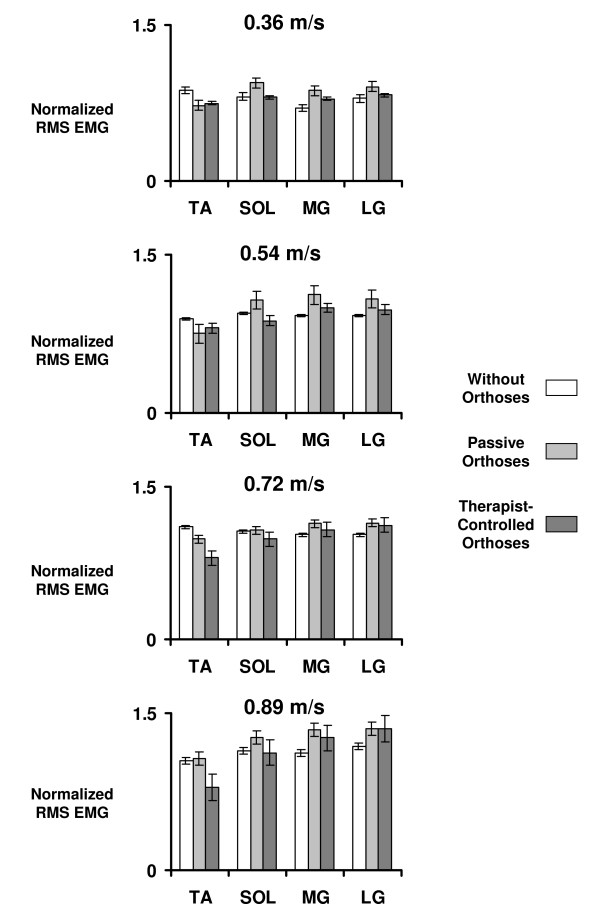
**Stance phase RMS EMG for without vs. passive vs. therapist-controlled orthoses across speeds**. Muscle activation amplitudes for tibilais anterior (TA), soleus (SOL), medial gastrocnemius (MG) and lateral gastrocnemius (LG). Data is from subjects with partial paralysis who walked with partial bodyweight support on a treadmill at (top to bottom) 0.36 m/s (five subjects), 0.54 m/s (five subjects), 0.72 m/s (four subjects) and 0.89 m/s (four subjects). Bars indicate mean ± standard error of the normalized average root mean square (RMS) EMG amplitude calculated during the stance phase for walking without-orthoses (WO), wearing orthoses unpowered or passive (PA) and wearing orthoses powered under pushbutton control by a therapist (TC).

Activation in five of the eight muscles studied was significantly higher when subjects walked with orthoses passive compared to when they walked without orthoses. Figure 4 shows the average normalized root mean square EMG of the ankle muscles over the stance phase of walking for each speed. Muscle activation was significantly higher in the passive-orthoses condition for medial gastrocnemius (18% higher) and lateral gastrocnemius (14% higher) (p < 0.05) (Table 3). For the knee extensor muscles, the stance phase RMS EMG for vastus medialis (10% higher), vastus lateralis (12% higher) and rectus femoris (10% higher) was greater in the passive-orthoses condition than in the without-orthoses condition (p < 0.05) (Table 3). There was no difference in activation for tibialis anterior, soleus or medial hamstrings (p > 0.05).

**Figure 5:**
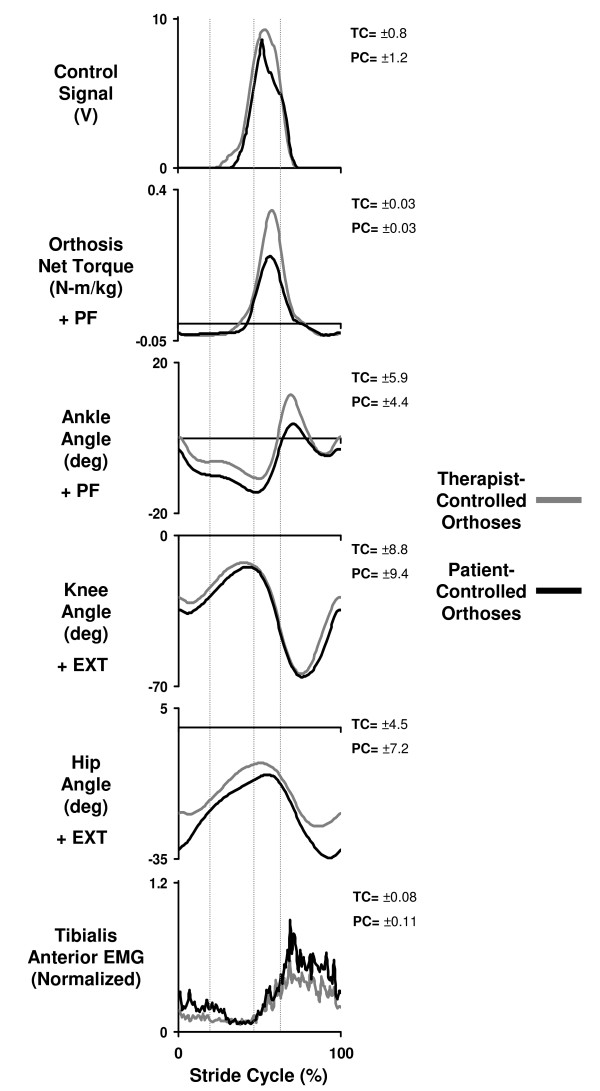
**Kinematics, kinetics and electromyography for therapist-controlled vs. patient-controlled orthoses**. Mean data for two subjects with incomplete spinal cord injury walking at 0.54 m/s wearing the orthoses powered under pushbutton control by a therapist (therapist-controlled orthoses) and powered under pushbutton control by themselves (patient-controlled orthoses). Stride cycles begin (0%) and end (100%) at heel strike. Double support phases are indicated by vertical lines. The average standard deviation over the stride cycle for each signal and each condition is reported to the right of each plot in units consistent with that signal.

Powered assistance under therapist control slightly decreased muscle activity in the soleus but not in medial or lateral gastrocnemius (Figure 2, Figure 4). Soleus RMS EMG decreased by 13% in the therapist-controlled condition compared to the passive condition (p < 0.05) (Table 3). Medial and lateral gastrocnemius RMS EMG decreased by 7% and 5%, respectively, in the therapist-controlled condition compared to the passive condition, but these differences were not statistically significant (p > 0.05) (Figure 4, Table 3).

Therapist-controlled powered assistance at the ankle joint had little effect on activation in the knee muscles. Medial hamstrings and vasti RMS EMG were not affected by therapist-controlled powered assistance (p > 0.05) (Table 3). Rectus femoris RMS EMG was 9% lower in the therapist-controlled orthoses condition compared to the passive-orthoses condition (p < 0.05) (Table 3).

### Patient-controlled vs. therapist-controlled orthoses

Two of the five subjects learned to use the pushbuttons to control the timing and amplitude of the orthoses assistance. The three other subjects practiced but were unable to learn the appropriate timing of the pushbuttons possibly due to the high level of cognitive attention and coordination required.

The amplitude and timing of the control signal was similar for the patient-controlled orthoses and therapist-controlled orthoses conditions. The two successful subjects generated a control signal with onset at 33.4 ± 2.2% of the gait cycle. The control signal reached a maximum 9.4 ± 0.3 V. This resulted in orthoses ankle plantar flexor torque onset at 43.5 ± 3.7% of the gait cycle. The timing of torque onset was not significantly different from the therapist-controlled orthoses condition (p > 0.05) (Figure 5).

The amplitude of orthoses torque assistance was lower for the patient-controlled orthoses condition compared to the therapist-controlled orthoses condition (p < 0.05) (Figure 5). The powered orthoses applied 0.33 ± 0.02 N-m/kg peak ankle plantar flexor torque near the end of the stance phase. This was ~0.07 N-m/kg lower than the therapist-controlled orthoses condition.

Joint kinematics were similar between the patient-controlled orthoses and therapist-controlled orthoses conditions with a few exceptions. The maximum ankle angle at push-off was ~5 degrees lower in the patient-controlled condition when compared to the therapist-controlled condition (p < 0.05) (Figure 5, Table 4). There were no significant differences at the knee or hip (p > 0.05) (Figure 5, Table 4). The stance time was 843 ± 62 ms in the patient-controlled condition. This was significantly different (~64 ms less) than the therapist-controlled condition (p < 0.05) (Table 4).

There were no significant differences in the activation of any muscles except for tibialis anterior. Tibialis anterior RMS EMG was 45% higher in the patient-controlled condition than therapist-controlled condition (p < 0.05) (Figure 5, Table 5).

## Discussion

The purpose of this study was to test the effects of robotic mechanical assistance at the ankle on joint kinematics and muscle activation patterns during walking by subjects with incomplete spinal cord injury. The robotic assistance resulted in ankle push-off kinematics similar to healthy walking at slower walking speeds and a slight reduction in muscle activation of the soleus but not the medial or lateral gastrocnemius across speeds.

Under therapist control, the therapist quickly learned to use pushbuttons to activate the orthoses, providing ankle torque with timing and amplitude similar to normal walking. In healthy subjects, triceps surae muscles normally develop active force from 10% to 60% of the stride cycle [41]. In our study, the therapist activated the orthoses so that they developed active force from 25% to 60% of the stride cycle (Figure 2). Peak plantar flexion torque in healthy subjects ranges from ~1.0–1.75 N-m/kg [42,43]. In this study the orthoses generated less peak torque (~0.4 N-m/kg), but bodyweight support (~38% unloading on average) decreased the mechanical loading of the limbs. Both forward and vertical work per stride are reduced in direct proportion to bodyweight unloading during walking [44]. Assuming that the same proportional reduction occurs in the peak ankle torque for healthy walking, we would expect peak plantar flexion torque to range from ~0.62–1.1 N-m/kg with ~38% bodyweight unloading. Therefore, the orthoses provided ~36–65% of the normal peak plantar flexion torque expected during walking with this level of bodyweight support. When the powered ankle-foot orthoses were worn by healthy subjects during walking without bodyweight support, they provided ~56% peak ankle plantar flexor torque [40]. It is important to highlight the possibility that adjustments by both the patient and therapist could result in the observed timing of ankle assistance. Future work could examine the transient behaviour of both the patient and therapist during the period leading up to when a steady cycle is established.

The torque assistance supplied by the powered ankle-foot orthoses improved ankle push-off kinematics at slower speeds. For healthy subjects walking at 0.54 m/s, ankle angle at push-off is ~10–12 degrees plantar flexion [45]. Ankle angle trajectories are not affected by bodyweight support levels below 75% so the support levels in this study should not have affected ankle kinematics [45]. A smaller ankle angle at push-off is typical of subjects with spinal cord injury due to limited ankle propulsion at the end of the stance phase [31]. As expected, our subjects were unable to reach normal plantar flexion when walking at 0.54 m/s without the orthoses (mean 4 deg) or with passive-orthoses (mean 1 deg). When subjects walked at 0.54 m/s with powered orthoses under therapist control, maximum ankle angle near push-off increased to a normal level (mean 12 degrees) (Table 2). The time of double support was significantly greater in the therapist-controlled condition (Table 2). Increased double support time can be an indication of reduced stability. Some subjects reported that they felt unstable in the active conditions. Perhaps training over multiple sessions could improve stability.

The powered orthoses were not as effective at increasing ankle push-off angle at higher walking speeds. For both healthy and subjects with spinal cord injury, ankle push-off angle increase as walking speed increases [31,45]. When our subjects walked without orthoses and with passive-orthoses, maximum ankle angle at push-off increased with speed as expected (Figure 3, Table 2). In contrast, there was a decrease in ankle angle at push-off with increasing speed when subjects walked with the powered orthoses. Two possible explanations are pneumatic actuator limitations and pushbutton control limitations. It is unlikely that the actuators caused the decline in ankle range of motion at faster speeds. A previous study using the powered orthoses on healthy subjects demonstrated ample force production and range of motion at faster walking speeds [40]. That study used footswitch controllers to activate the pneumatic actuators automatically during stance rather than handheld pushbuttons. It is possible that faster walking speeds required more precise timing of the pushbuttons to activate the artificial muscles. In the current study, the stance phase duration decreased from 1.26 seconds to 0.74 seconds as walking speed increased from 0.36 m/s to 0.89 m/s. Shorter stance duration results in a smaller time period to activate the orthoses assistance. Small absolute errors in timing may become significant at fast speeds because of increased relative error with respect to the stride cycle. To reduce the possibility for errors in timing future designs could automatically trigger assistance during the stride with a footswitch.

An important result of this study was that mechanical assistance at the ankle joint did not substantially reduce muscle activation in the plantar flexors. Sinkjaer et al. [46] used a mechanical device to quickly perturb the ankle joint during walking and found a clear plantar flexor muscle response to imposed loading in healthy subjects. When the ankle was forced into rapid plantar flexion, soleus activity was reduced by up to ~50% [46]. They concluded that muscle spindle group II afferents and Golgi tendon organ group Ib afferents were responsible for these modifications in muscle recruitment. In our study, mechanical assistance caused only a 13% decrease in soleus muscle activation during stance (Figure 4, Table 2). An important difference between the perturbation study and our study is the rate of the ankle unloading. In the perturbation study, the ankle joint was rapidly unloaded at approximately 440 N-m/s. In our study, the ankle joint was unloaded at approximately 85 N-m/s. This rate is more characteristic of normal plantar flexion torque development [40]. Studies that use full body unloading are more analogous to the unloading in our study because the bodyweight support is nearly constant (i.e. unloading rate ~0 N-m/s). Ferris et al. found a 10–15% reduction in soleus muscle activation with 50% bodyweight support in healthy subjects [47]. Harkema et al. [12] reported similar reductions in soleus muscle activation in subjects with incomplete spinal cord injury.

When subjects walked with passive-orthoses, muscle activity during stance increased in five of the eight muscles compared to the without-orthoses condition. There are several factors that may have led to this result. The elastic bands providing dorsiflexion torque on the orthoses could have influenced the stance phase activation by resisting plantar flexion. The orthosis added mass to the lower limb, but this should have only affected swing phase muscle activation rather than stance phase muscle activation. The orthoses limited ankle joint motion to dorsiflexion/plantar flexion and stabilized the other degrees of freedom of the ankle joint. It is possible that increased joint stability in off axis motion could lead to a decrease in neural inhibition to the plantar flexors [48-50]. Future studies should examine these possibilities in greater detail as they could potentially have clinical implications for improving gait of individuals with spinal cord injury.

Although we expected that subjects would be able to use the pushbuttons to control the orthoses, most were not able to do so. Three of five subjects were unable to adequately control the orthoses with the pushbuttons. Cognitive deficits, sensory impairment, spasticity and muscle weakness are all factors common in spinal cord injury populations that could contribute to difficulties in learning to coordinate an assistive device. Based on feedback from the subjects, manipulating pushbuttons while attempting to walk required too large of a cognitive effort. Even the two subjects who could control the orthoses themselves did not match the performance of the therapist. Both orthoses torque and ankle angle at push-off were reduced for patient-controlled compared to therapist-controlled conditions (Figure 5, Table 4). These findings suggest that future robotic rehabilitation devices designed to place the patient in the control loop need to simplify the controller interface or somehow reduce cognitive demand of the patient.

## Conclusion

Robotic assistance at the ankle can improve push-off kinematics in individuals with incomplete spinal cord injury without large decreases in muscle activation amplitudes. The therapist-controlled trials suggest that it is feasible for robotic rehabilitation devices to incorporate observer-mediated control. It might also be possible to improve the consistency of the assistance by using automatic triggering (eg. a footswitch). The patient-controlled trials indicate that self-operated robotic rehabilitation devices may require higher-level controllers that allow off-line adjustments over long time scales (i.e. every third step vs. every single step) and reduce patient cognitive effort. This study quantifies changes in kinematics and muscle activation patterns due to powered ankle assistance within a single test session following a single session of training. Future studies are needed to track changes over multiple sessions and assess long-term training effects. As well, studies are needed to test whether training with robotic assistance at the ankle can improve functional walking ability in the incomplete spinal cord population.

## Competing interests

The author(s) declare that they have no competing interests.

## Authors' contributions

GSS recruited subjects, managed all data collections, completed all data analysis and drafted the manuscript. AD recruited subjects, assisted with data collections and edited the manuscript. DPF conceived of the study, provided expert guidance on experimental design, assisted with data collections and helped edit the manuscript. All authors read and approved the final manuscript.
